# Ischemic Colitis in a Young Female Following Herbal Supplement Ingestion

**DOI:** 10.7759/cureus.45315

**Published:** 2023-09-15

**Authors:** Lorraine I Chong Tai, Syed Ahmed, Rajiv R Chokshi

**Affiliations:** 1 Internal Medicine, Broward Health Medical Center, Fort Lauderdale, USA; 2 Internal Medicine, Broward General Medical Center, Fort Lauderdale, USA

**Keywords:** ischaemic colitis, colitis, young population, herbal supplements, ischemic colitis

## Abstract

Ischemic colitis is typically considered a disease of the elderly, given their atherosclerotic risk factors and other comorbidities. Ischemic colitis in the younger population is considered very uncommon. We present an interesting case of a young female presenting with abdominal pain and hematochezia found to have ischemic colitis on pathological biopsy results after a colonoscopy. She was an otherwise healthy female with no medical problems prior to her hospitalization but endorsed recent use of a bitter herbal tea to relieve her symptoms. Here, we review potential causes of ischemic colitis, including herbal remedies, which have been linked to causing hypercoagulable and hypotensive states.

## Introduction

Ischemic colitis has an estimated annual incidence of 15.6 to 17.7 per 100,000 [1]. The risk of ischemic colitis is higher in females and increases in those 65 years and older [2]. Ischemic colitis is often seen in patients with comorbidities, including hypertension, tobacco use, vascular diseases, diabetes mellitus, hyperlipidemia, and atrial fibrillation [3]. Rare cases in younger patients have been associated with oral contraceptive use, coagulopathies, vasculitis, illicit drug use, and long-distance running in younger patients [4]. Other causes might include vasoconstrictive, anticoagulant, or hypotensive effects from some herbal supplement use, but clinicians are often left unable to identify a potential etiology [5]. We describe a case of ischemic colitis in a 40-year-old woman with no apparent risk factors for ischemia, with the suspected cause being the recent ingestion of an herbal tea.

## Case presentation

A 40-year-old female of Haitian descent presented to the emergency department with complaints of severe diffuse, sharp, and constant abdominal pain that began the night prior to presentation. She reported one episode of large-volume hematochezia with loose stool the day prior. She endorsed symptoms of nausea, three episodes of non-bloody emesis, poor oral intake, fever, chills, rhinitis, and a dry cough five days earlier that resolved after one day. She denied constipation, changes in diet, recent travel, recent sick contacts, weight loss, or taking any medications. She denied any illicit drug use, history of abdominal surgeries, and any history of tobacco use, and her alcohol intake was minimal. Family history was unremarkable for any history of inflammatory bowel disease (IBD), gastrointestinal (GI) malignancies, or coagulopathies. She had no history of miscarriages and carried two pregnancies successfully to term. She denied any history of oral contraceptive pill (OCP) use but endorsed recently taking an herbal bitter tea of unknown ingredients to relieve her symptoms. Her last menstrual period (LMP) was two weeks prior with regular menstrual cycles.

**Figure 1:**
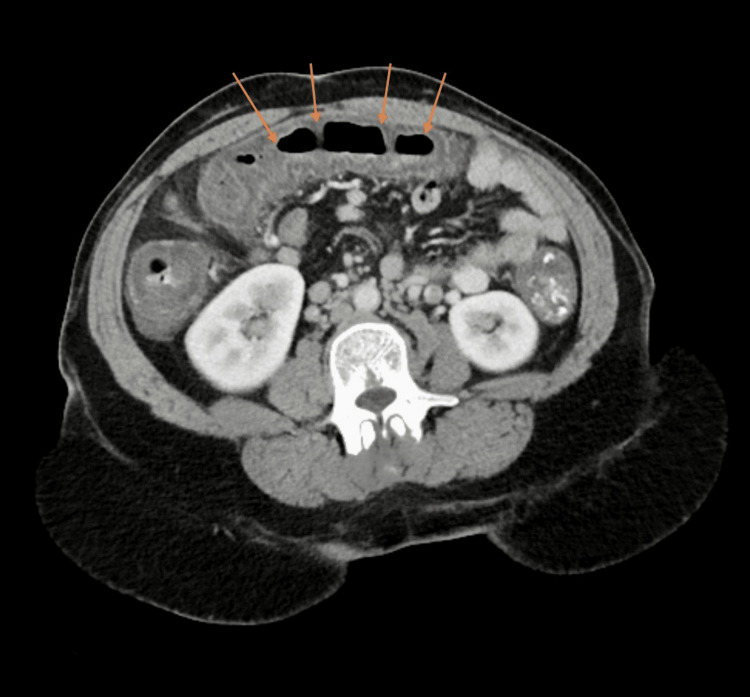
Contrast enhanced CT of the abdomen/pelvis showing ​​diffuse thickening of the transverse colon (arrows), representing colitis

**Figure 2:**
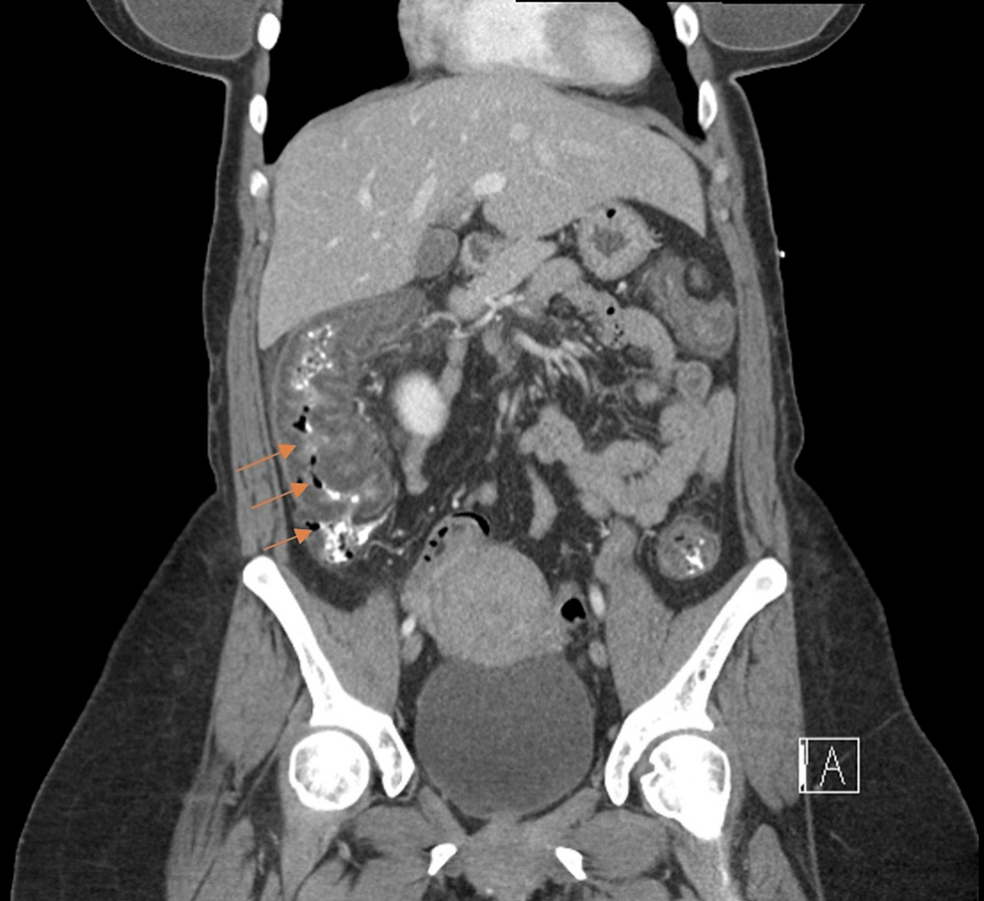
Contrast enhanced CT of the abdomen/pelvis showing diffuse thickening of the ascending colon (arrows), representing colitis

**Figure 3:**
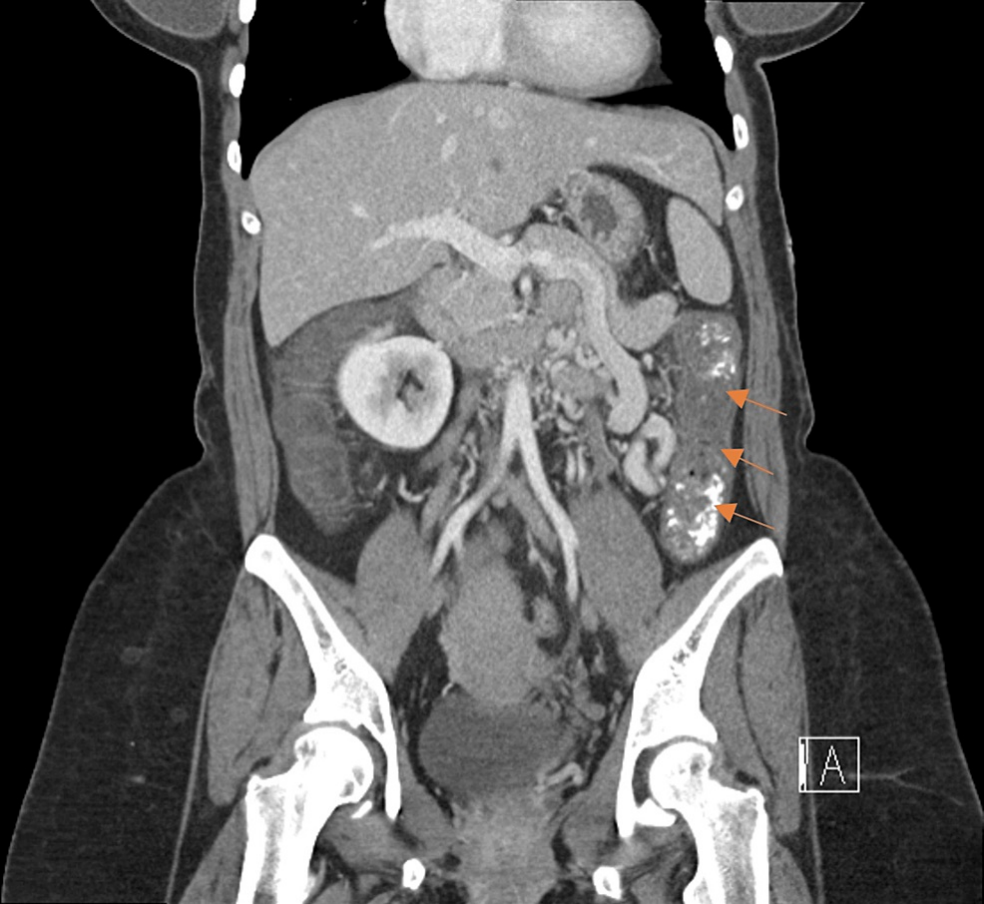
Contrast enhanced CT of the abdomen/pelvis showing diffuse thickening of the sigmoid colon (arrows), representing colitis

Upon presentation, she was afebrile and her vitals were stable. Abdominal examination was soft, with tenderness to palpation in the lower abdomen without guarding, no rebound tenderness or signs of peritonitis, and non-distended with normal bowel sounds. Gross blood was appreciated on rectal examination. In the emergency department, she was given 12 mg of morphine without symptomatic relief. Initial laboratory studies showed no leukocytosis and stable hemoglobin at 14.0 g/dL (Table 1). Computerized tomography (CT) of the abdomen/pelvis with Intravenous (IV) contrast revealed diffuse thickening of the colon, beginning at the sigmoid and extending into the ascending colon, most likely infectious versus ischemic colitis (Figures 1-3).

**Table 1:** Laboratory investigations

Lab results (units and reference range)	Results
Hemoglobin (14.0-18.0 g/dL)	14.0
White blood cell count (4.80-11.80 x 10^3^/mcL)	7.08
Platelets (150-450 x 10^3^/mcL)	225
aPTT (25.1-36.5 seconds)	29.4
INR	1.1
PT (12.0-14.4 seconds)	14.6
BUN (6-23 mg/dL)	5
Creatinine (0.70-1.20 mg/dL)	0.7
ESR (0-20 mm/h)	5
CRP (0.3-1.0 mg/dL)	0.58, 6.59 (repeat on hospital day 2)
Total cholesterol (0-200 mg/dL)	117
Triglycerides (<150 mg/dL)	54
HDL cholesterol (35-80 mg/dL)	64
LDL cholesterol (<100 mg/dL)	42
HgA1C (<5.7%)	4.5
Lactic acid (0.5-2.2 mmol/L)	4.8
Fecal occult blood test	Positive
Treponema syphilis	Non-reactive
HIV antibody/antigen combo	Non-reactive
ANCA	Negative
ANA	Negative

Standard stool cultures, clostridium difficile antigen, and Gastrointestinal Biofire panel were all negative. Despite these findings, she was empirically treated for an infectious cause with Flagyl and given IV fluids. The fecal occult blood test was positive. Lactic acid remained within normal limits throughout hospitalization.

**Figure 4:**
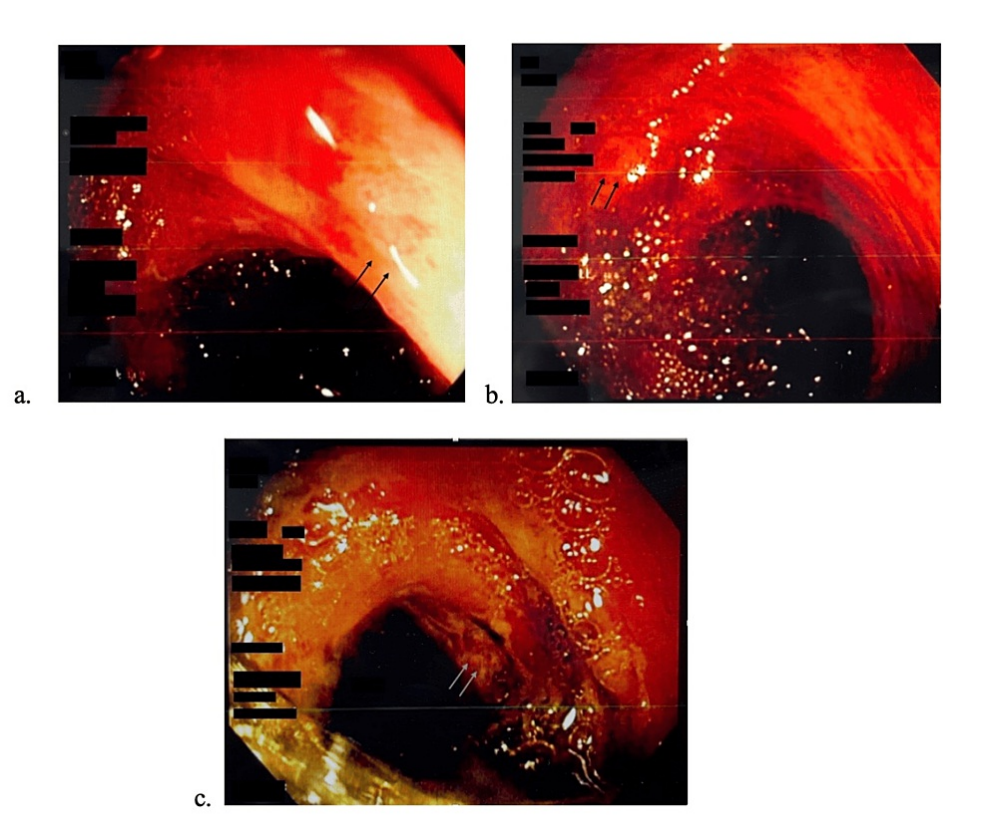
Colonoscopy results of erythematous mucosa indicating colitis (a) Distal ascending colon with arrows indicating edema of the colon (b) Transverse colon with arrows indicating ulceration (c) Proximal descending colon with arrows indicating ulceration

A surgical consult was obtained for intractable abdominal pain, after which a colonoscopy was recommended to evaluate for potential IBD. She underwent a colonoscopy that showed diffuse colitis, edema, congestion and ulceration of the distal ascending colon, transverse colon, and proximal descending colon suggestive of ischemic colitis (Figures 4a-4c). Biopsy results of the left colon were significant for colonic glands with a withered appearance, hyalinization of the lamina propria, mucosal hemorrhage, and detached surface epithelium present, confirming ischemic colitis.

Vasculitis workup included testing for Treponema syphilis, human immunodeficiency virus (HIV), antinuclear antibody (ANA), and antineutrophil cytoplasmic antibodies (ANCA), which were all unremarkable. Her lipid panel was also unremarkable. Coagulation studies revealed a normal international normalized ratio (INR) and partial thromboplastin time (PTT) with a slightly prolonged prothrombin time (PT) of 14.6 seconds. Erythrocyte sedimentation rate (ESR) and C-reactive protein (CRP) were initially normal, but repeated CRP on day 2 of hospitalization was elevated at 6.59 mg/dL (Table 1). Hemoglobin remained stable throughout hospitalization, and she did not require any transfusions. She was recommended to stop drinking her herbal tea and to follow up with a gastroenterologist outpatient with a repeat colonoscopy recommended in six months. A hypercoagulable workup was planned, but unfortunately, the patient left before these laboratory studies were completed and failed to follow up.

## Discussion

Approximately 90% of ischemic colitis occurs in patients over 60 years old [6]. Ischemic colitis in the younger population is considered rare, and therefore, there is often a low clinical suspicion for ischemic colitis over infectious or inflammatory causes. Several potential risk factors have been identified in younger patients. One case report has described ischemic colitis as a result of herbal product use with vasoconstrictive properties in a young female [4].

The herbal supplement *Momordica charantia*, otherwise known as bitter melon, has been proposed to have an anticoagulant-like effect and may even cause hypotension. One study found that *M. charantia* was not associated with a significant reduction in either systolic or diastolic blood pressure, and there was a significant hypotensive effect in younger adults [7]. Therefore, the possible etiology behind the ischemic colitis in our patient could be secondary to reduced blood flow to the colon in the setting of hypotension vs. anticoagulant effects of *M. charantia*. Manjappa et al. found that *M. charantia* seed extract exhibits a strong anticoagulant effect due to its fibrin clot-hydrolyzing properties [8]. Although the exact nature of the herbal supplement is unknown to our patient, given her description of its bitter taste, the known side effects, and its popularity among the Haitian population, it is quite likely that *M. charantia* was a component of her herbal tea. 

Other etiologies linked to ischemic colitis include OCP use and estrogen. Although the exact pathophysiology remains unknown, it has been proposed that it may be related to estrogen increasing the risk of thrombosis [9]. Ischemic colitis overall has also been found to have a strong female predominance, but in a retrospective study of 39 young adults (<50 years of age) diagnosed with ischemic colitis, almost half of the patients did not have an identifiable etiology [5]. Antiphospholipid antibodies and Factor V Leiden mutations were found 10 times more frequently in patients with ischemic colitis [10]. The 506 Q allele of the factor V (FV) 506 RQ (Leiden) mutation and the mutant 4G allele of plasminogen activator inhibitor (PAI) polymorphism were found to be significantly higher in the younger population with ischemic colitis [8]. However, it seems that an inherited coagulopathy is less likely in this patient, given her history of two successful pregnancies without any history of miscarriages. A hypercoagulable workup is likely clinically indicated for this patient. 

Findings such as elevated lactate, metabolic acidosis, and significant base deficit may be present in cases of severe ischemia and/or necrosis, but this is generally a late sign [1]. As described in this case report, the left colon is the predominant location of ischemia in approximately 75% of patients with injury typically occurring in the “watershed” areas of the splenic flexure (Griffith point) and sigmoid colon (Sudeck point) [6,11].

CT imaging of the abdomen is the most helpful in initial assessment as it can exclude other sources of abdominal pain, suggest a location and source of ischemia, and identify complications associated with more advanced disease [1]. However, colonoscopy remains the diagnostic test of choice to evaluate the degree of ischemia [11]. Obtaining angiography is typically not helpful in diagnosing ischemic colitis, as most cases involve transient small vessel hypoperfusion. Angiography would be most helpful in cases where an embolus is suspected [1].

Regardless of age, management is often conservative with IV fluid, bowel rest, correction of underlying conditions, and antibiotic therapies [12]. If conservative management is unsuccessful within 24 to 48 hours, repeat colonoscopy or imaging of the mesenteric vasculature with CT angiography is then indicated to reevaluate the severity and degree of the disease. Alarming signs, including increasing abdominal tenderness with guarding and rebound tenderness, fever, uncontrollable bleeding, and paralytic ileus, indicate severe disease for which urgent laparotomy and removal of the necrotic part of the colon would be necessary [13].

Overall, the prognosis is favorable for younger patients. Mortality is low and typically increases with age and comorbidities [14].

## Conclusions

Potential etiologies of ischemic colitis in young people are not well studied. Despite the low clinical suspicion of ischemic colitis in this age group, this case report emphasizes the importance of taking a thorough history in investigating for less common etiologies. In younger patients with confirmed ischemic colitis, a thorough review of their past medical history, medications, and supplements may be helpful in identifying other risk factors. Given the patient’s history of recent herbal supplement use and lack of other risk factors, we believe that her ischemic colitis was likely secondary to her herbal tea. A hypercoagulable workup is also likely warranted in these cases to rule out an inherited versus an acquired hypercoagulable state from medications or supplements. 

## References

[1] Washington C, Carmichael JC (2012). Management of ischemic colitis. Clin Colon Rectal Surg.

[2] Higgins PD, Davis KJ, Laine L (2004). Systematic review: the epidemiology of ischaemic colitis. Aliment Pharmacol Ther.

[3] Chang HJ, Chung CW, Ko KH, Kim JW (2011). Clinical characteristics of ischemic colitis according to location. J Korean Soc Coloproctol.

[4] Ryan CK, Reamy B, Rochester JA (2002). Ischemic colitis associated with herbal product use in a young woman. J Am Board Fam Pract.

[5] Preventza Preventza, O.A. O.A., Lazarides Lazarides, K. & Sawyer, M.D M.D (2001). Ischemic colitis in young adults: a single-institution experience. J Gastrointest Surg.

[6] Theodoropoulou A, Koutroubakis IE (2008). Ischemic colitis: clinical practice in diagnosis and treatment. World J Gastroenterol.

[7] Jandari S, Ghavami A, Ziaei R (2020). Effects of Momordica charantia L. on blood pressure: a systematic review and meta-analysis of randomized clinical trials. Int J Food Prop.

[8] Manjappa B, Gangaraju S, Girish KS (2015). Momordica charantia seed extract exhibits strong anticoagulant effect by specifically interfering in intrinsic pathway of blood coagulation and dissolves fibrin clot. Blood Coagul Fibrinolysis.

[9] Jones B, Alpert L, Reddy G, Coronel E (2016). A case report of ischemic colitis in a young woman on combined oral contraceptives: 1356. Am J Gastroenterol.

[10] Koutroubakis IE, Sfiridaki A, Theodoropoulou A, Kouroumalis EA (2001). Role of acquired and hereditary thrombotic risk factors in colon ischemia of ambulatory patients. Gastroenterology.

[11] FitzGerald JF, Hernandez Iii LO (2015). Ischemic colitis. Clin Colon Rectal Surg.

[12] Xu Y, Xiong L, Li Y, Jiang X, Xiong Z (2021). Diagnostic methods and drug therapies in patients with ischemic colitis. Int J Colorectal Dis.

[13] Misiakos EP, Tsapralis D, Karatzas T (2017). Advents in the diagnosis and management of ischemic colitis. Front Surg.

[14] Cosme A, Montoro M, Santolaria S (2013). Prognosis and follow-up of 135 patients with ischemic colitis over a five-year period. World J Gastroenterol.

